# An evaluation of randomized controlled trials on nutraceuticals containing traditional Chinese medicines for diabetes management: a systematic review

**DOI:** 10.1186/s13020-019-0276-3

**Published:** 2019-11-29

**Authors:** Junnan Shi, Hao Hu, Joanna Harnett, Xiaoting Zheng, Zuanji Liang, Yi-Tao Wang, Carolina Oi Lam Ung

**Affiliations:** 1 State Key Laboratory of Quality Research in Chinese Medicine, Institute of Chinese Medical Sciences, University of Macau, Taipa, Macao; 2 The University of Sydney School of Pharmacy, Faculty of Medicine and Health, The University of Sydney, New South Wales, Australia

**Keywords:** Nutraceuticals, Supplements, Traditional Chinese medicine, TCM, Blood glucose, Diabetes, Randomized clinical trial, RCT

## Abstract

**Background:**

Nutraceuticals containing traditional Chinese medicine (TCM) are promoted for use in the management of diabetes. The evidence to support such use is largely unknown. This study aimed to summarise and evaluate the literature reporting the results of randomized controlled trials (RCTs) investigating the effects of nutraceuticals in people living with diabetes.

**Methods:**

Literature from four electronic databases (PubMed, Scopus, CINAHL and Web of Science) was searched following PRISMA guidelines to yield RCT publications on nutraceutical for diabetes management published since 2009. The quality of reporting was assessed using the CONSORT 2010 checklist statement. Risk-of-bias for each study was assessed using the Cochrane risk of bias tool.

**Results:**

Out of 1978 records identified in the initial search, 24 randomized, double/triple-blinded, controlled trials that investigated the effect of nutraceuticals covering 17 different TCM herbs for diabetes management were selected. Participants included people who were diabetic (n = 16), pre-diabetic (n = 3) or predisposed to diabetes (n = 5). Sample sizes ranged between 23 and 117 for 2 arms, or 99–165 for 3 arms. Comparisons were made against placebo (n = 22), conventional medicine (n = 1), or regular diet (n = 1) for a duration between 4 and 24 weeks. All but one study tested the effect on fasting blood glucose levels (n = 23) or glycated haemoglobin levels (n = 18), and/or postprandial 2-h blood glucose levels (n = 4) as the primary outcomes. Nineteen studies reported some statistically significant reductions in the respective measures while 5 studies showed no effect on primary or secondary outcomes. None of the included studies met all the criteria for the CONSORT guidelines. Incomplete reporting about randomization and blinding, and a lack of ancillary analyses to explore other influential factors and potential harms associated with the use were repeatedly noted. Based on the Cochrane risk-of-bias tool, 19 studies were deemed to have a high risk of bias mainly attributed to sponsor bias.

**Conclusions:**

There is some evidence to suggest positive clinical outcomes in response to the administration of a range of nutraceuticals containing TCM in the management of diabetes. However, these results must be interpreted with caution due to the overall low quality of the trials.

## Background

The prevalence of diabetes has been increasing worldwide. With population growth, aging and urbanisation, and related lifestyle changes, it is estimated that diabetes affects 439 million adults aged from 20 to 79 years, accounting for 7.7% of the global population, especially in developing countries [1]. At the same time, diabetes itself is a multi-morbid metabolic disorder, and in the long-term it can cause damage and dysfunction of different organs, leading to decreased quality of life and increased mortality [2]. These factors would have a significant economic impact on global health care. Based on the current pathological and physiological understanding of diabetes, receiving insulin therapy and oral hypoglycaemic agents are the most common medical treatment for the disease [3, 4].

Traditional Chinese medicine (TCM) is an important form of complementary medicine. Numerous studies have reported that some traditional herbs and plants have a significant effect on many aspects of managing diabetes, such as *Momordica charantia* L. (*Cucurbitaceae*), *Trigonella foenum graecum* L. (*Leguminosae*) and also some active components like plant polyphenols [3–6]. As anti-diabetic medicinal plants tend to be more affordable and reportedly have less serious side-effects than synthetic drugs, people are increasingly willing to choose TCM as a complementary form of diabetes self-management [7, 8]. According to China Customs statistics, the total value of TCM import and export reached 4.6 billion USD in 2016, accounting for 4.45% of the total value of the import and export of Chinese pharmaceutical products [9]. However, high policy thresholds and trade barriers in many developed countries have hindered the process of internationalization of TCM. For instance, in the countries of European Union, herbal medicines for human use must follow the latest legislations and different requirements before they are authorized. Another marketing pathway is for eligible herbal medicines to be marketed as dietary supplements which are subject to the food regulations [10–12].

Nutraceuticals and dietary supplements are widely used, and a survey from the Centers for Disease Control and Prevention in United States showed that 20% of adults used supplements that contained at least one plant ingredient [13]. The terms “nutraceuticals”, “dietary supplements” and “functional foods” are often been used interchangeably. According to the definitions given by the Canadian health department and that of the United States and China [14–16], adding the purpose of this review, “nutraceuticals” are defined as “products composed of one or more substances, which have physiological benefit or provide protection against chronic diseases, are isolated from foods, generally sold in medicinal forms (such as pills, powder potions), and not usually associated with food, especially not including fortified foods”. Although nutraceuticals containing TCM are promoted for their positive effects in the management of diabetes, such as reducing the blood glucose levels, their efficacy is largely unknown [17]. However, some nutraceutical formulations containing specific TCM have been clinically tested to verify their effectiveness in the management of diabetes including the effects of ginger supplements on insulin resistance and cinnamon supplements for glycaemic control in patients with type 2 diabetes mellitus (T2DM) [18, 19]. To date, the literature reporting randomized controlled trials (RCTs) involving nutraceuticals containing TCM has not been systematically reviewed. Therefore, the aim of this review is to summarise and evaluate the quality of studies reporting the results of randomised controlled trials investigating the effects of nutraceuticals containing TCM in the management of diabetes.

## Methods

The systematic review conforms to the Preferred Reporting Items for Systematic Reviews and Meta analyses (the PRISMA statement) and utilizes the Consolidated Standards of Reporting Trials Statement (the CONSORT statement), and the Cochrane Collaboration’s Risk of Bias tool for evaluating the quality of the studies included the review.

### Eligibility criteria

#### Types of studies

Randomized, double or triple-blinded, controlled trials evaluating the effects of nutraceuticals containing TCM in diabetes and published in English were included. Nutraceuticals that contained TCM listed in the Chinese Pharmacopoeia were eligible for inclusion in this review [20].

#### Participant characteristics

Studies that included patients regardless of the gender, age, or race/ethnicity were eligible. The target populations included people living with diabetes, pre-diabetes or people predisposed to diabetes. Diabetes was diagnosed clinically according to the American Diabetes Association (ADA) diagnostic criteria, including Type 1 diabetes mellitus (T1DM), Type 2 diabetes mellitus (T2DM) and other specific types of diabetes [21]. ADA reintroduced pre-diabetes to cover impaired glucose tolerance (IGT) and impaired fasting glucose (IFG) in 2005, however, any definition of pre-diabetes limited to IGT and/or IFG does not include other risk factors for diabetes [22, 23]. People who are predisposed to diabetes are affected by many increased risks, such as obesity, hypercholesteremia, metabolic syndrome (MetS), which have been evidenced from several studies to increase the risk of diabetes [24, 25].

#### Types of interventions

Patients in the control group were treated with placebo or/and conventional medicines or in addition to their regular diet. Patients in the treatment group were given nutraceuticals containing TCM in addition to the conventional medicines. Details of both the placebo and nutraceutical were required, including the TCM ingredients, formulation composition, extraction technology and any declarations of financial support.

#### Outcome measures

Studies including three widely accepted diagnostic criteria for diabetes were eligible: fasting blood glucose levels (FBG), postprandial 2-h blood glucose levels (PBG), and glycated haemoglobin (HbA1c) [26–29]. Studies reporting other outcomes such as fasting insulin, postprandial insulin, insulin sensitivity, the level of Homeostatic Model Assessment for Insulin Resistance index (HOMA-IR), the quantitative insulin sensitivity check index (QUICKI), and β-cell function were also eligible for inclusion [30, 31].

#### Quality assessment methods

The quality of the studies included was assessed using the 24-item version of the Consolidated Standards of Reporting Trials statement (CONSORT) [32]. The 2010 checklist provides a set of guidelines that may be used to identify the strengths and weaknesses of clinical trials for both pharmacological and non-pharmacological treatments. Two authors (JS, COLU) independently assessed the risk of bias for each article included, based on the 12 criteria recommended by the Cochrane Review Group [33]. Disagreements were settled through discussion or consultation with the corresponding authors.

### Search methods

Four electronic databases, including PubMed, Scopus, CINAHL and Web of Science were searched for RCT studies evaluating nutraceuticals containing TCM for diabetes management for the period 2009 until February 1, 2019. Search terms included: (nutraceutical* OR supplement*) AND (diabetes OR glucose OR insulin*) AND (clinical OR trial*). The PubMed database has the special medical terminology “MeSH”, so the terms “Glucose Metabolism Disorders [Mesh]” and “Dietary Supplements[MeSH]” were added to the search in this database to ensure that results were not missed.

### Exclusion criteria and screening

Abstracts and full text articles were rigorously reviewed for meeting the inclusion criteria. Non-TCM-related RCTs were screened for inclusion in three stages during the title retrieval: (1) review, meta-analysis, protocol were not included; (2) vitamin, mineral, fortified food and beverage, probiotic and prebiotic were also not included; (3) herbal ingredients not listed in the Chinese Pharmacopoeia were not included. In addition, observational studies that were limited by heterogeneity or reliance on self-reported nutritional data were not included.

### Data collection and analysis

#### Selection of studies

Two review authors (JS, COLU) independently screened the titles and abstracts identified in the search for meeting the inclusion criteria outlined above. Full texts of potentially relevant articles were retrieved for detailed assessment. The CONSORT and Cochrane evaluations were independently implemented by two authors (JS, COLU) in accordance with the guidelines, and discrepancies were discussed and resolved by agreement or consultation with 2 others author (HH, YTW).

#### Data extraction and management

Data were extracted based on study characteristics that included patients, methods, interventions and outcomes, into a standardised data extraction form. Reasons for the exclusion of studies were recorded. For eligible studies, two review authors (JS, COLU) extracted data, evaluated information independently and any disagreements were resolved by discussion, or by involving a third author.

#### Data synthesis and analysis

References were categorsied and filed using Endnote X9 and data extracted and categorized using Excel 2013. Data was extracted for primary outcomes (FBG, PBG, and HbA1c levels) and secondary outcomes (fasting insulin, postprandial insulin, insulin sensitivity, HOMA-IR, QUICKI, and β-cell function). Treatment effect was estimated with mean difference in the final value of FBG or PBG between the intervention and the placebo groups. The inverse variance-weighted method was used for the pooling of mean difference and the estimation of 95% confidence interval; the significant level was set at *P *<* 0.05* [34].

All included studies were assessed for compliance with the 2010 guidelines of the CONSORT statement. To measure compliance, a grading system was devised for each criterion, where the reviewer gave a score of “0” if the item was not present at all, a “1” if the feature was partially present, such as some aspects of the CONSORT item were missing or unclear, and a “2” if the item was present and clear. By applying the CONSORT criteria to all relevant sections of each study, an overall summary of the study’s quality as a clinical trial was produced. The evaluation method was checked for validity and consistency by 3 co-authors [35].

The assessment using the Cochrane risk-of-bias tool also has its own judging criteria. The results of each items were divided into three categories: “Low risk of bias”, “Unclear risk of bias” and “High risk of bias”. All criteria are suggested in the Cochrane guidelines. At the same time, this was also be checked by 3 co-authors [36].

## Results

### Study selection

**Fig. 1:**
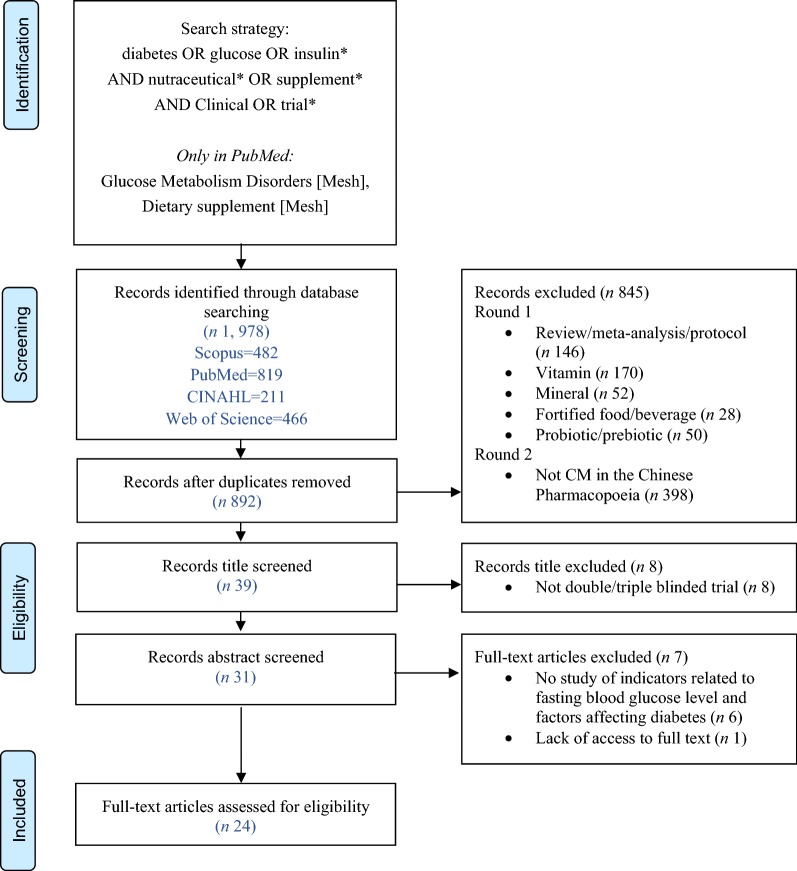
Prisma 2009 flow-chart summary of systematic review search process

We identified 892 potentially relevant articles from 1978 initial records after duplicates were removed. An additional 845 records were excluded during screening or a range of reasons: review or meta-analysis or protocol articles (n = 146); vitamin (n = 170); mineral (n = 52); fortified food and beverage (n = 29); probiotic and prebiotic (n = 50); or not TCM (n = 398); crossover trials (n = 2), observational clinical trials (n = 1) or single-blinded trials (n = 5); did not include biomarkers of diabetes (n = 6) and 1 article had a lack of access to full text. No additional eligible studies were found after manually searching the four electronic databases. Ultimately, 24 eligible studies, involving numbers of the participants between 23 and 165, and study durations from 4 to 24 weeks, were identified. The screening process is summarized in a flow diagram that followed the Prisma guidelines (Fig. 1).

### Study characteristics

The 24 studies were all published between 2009 and February 1, 2019 (see Table 1). All 1742 participants were from 11 different areas and countries. Among them, 644 were male and 900 were female (two trials did not report the number of patients according to gender). The study sample size ranged between 23 and 117 for 2 arms, or 99 to 165 for 3 arms. Seven RCTs had a small sample size of less than 50, 11 RCTs had a sample size between 50 and 100, and the other 6 RCTs ranged in sample size between 100 and 200.

**Table 1:** The effect of nutraceuticals containing TCM on managing diabetes through RCT

Chinese medicine	First author	Country	Design	Other ingredients	Relevant inclusion criteria	Subjects	Interventions	Relevant outcomes	Results
Cinnamon	Akilen et al. [37]	UK	Prospective, double-blind, placebo-controlled RCT	Starch powder	T2DMs, age ≥ 18 years, two consecutive FBG ≥ 7.0 mmol/L, HbA1c ≥ 7%, oral hypoglycaemic agents	*n* 58 (F = 33 and M = 25) Age = 54.43 (_SD_ 12.53) years (placebo), 54.90 (_SD_ 10.14) years (cinnamon)	Duration = 12 weeks Placebo or 2 g (500 mg × 4) cinnamon powder daily	Primary = FBG and HbA1c	There was a significant reduction in FBG compared to baseline in the cinnamon group but the changes were not significant when compared to placebo group (*P *=* 0.318*, changes = 14.0 (_SD_ 33.0) mg/dL), the mean HbA1c was significantly decreased (*P *< 0.005*, changes = 0.36 (SD 0.90) %) in the cinnamon group compared with placebo group
	Sharma et al. [38]	India	Prospective, double-blind, placebo-controlled RCT	N/A	Age ≥ 30 years, FBG level between 140–400 mg/dL, standard diet and exercise for 1 month	*n* 58 (F = 56 and M = 94)	Duration = 3 months Two arms: (1) 3 g/day dose of cinnamon as a 1 g capsule (2) 6 g/day of cinnamon as a 2 g capsules	Primary = FBG and HbA1c	There was a significant reduction in FBG (3 g *P *< 0.001*, 6 g *P *< 0.01*) and HbA1c (3 g *P *< 0.005*, 6 g *P *< 0.001*) level in both groups
	Mirfeizi et al. [39]	Italy	Multicenter stratified, triple‐blind, placebo-controlled RCT	Caucasian whortleberry (*Vaccinium arctostapphylos L*.), starch	T2DMs, HbA1c > 7% and FBG ≥ 140 mg/dL despite the oral blood glucose-lowering agents	*n* 102 (F = 79 and M = 23) Age = 55 (_SD_ 10) years (placebo), 52 (_SD_ 13) years (cinnamon), 55 (SD 10) years (whortleberry)	Duration = 3 months Placebo or (1) cinnamon supplements of 1 g/day, (2) whortleberry supplements of 1 g/day	Primary = FBG, PBG and HbA1c Secondary = Fasting insulin and HOMA-IR scores	There was a significant decrease in FBG (cinnamon *P *< 0.006*, whortleberry *P *< 0.002*), PBG (cinnamon *P *< 0.003*, whortleberry *P *< 0.001*) and HbA1c (cinnamon *P *< 0.010*, whortleberry *P *< 0.007*) level in both groups, Fasting insulin and HOMA-IR also showed a significant effect (*P *< 0.05*)
Cinnamon	Gupta Jain et al. [40]	India	Parallel, triple-blind, placebo-controlled RCT	Wheat flour	Metabolic syndrome, stable	*n 116* (*F = 52 and M = 64*) *Age = 45.1* (*SD 8.4*) *years* (*placebo*), *44.3* (*SD 7.2*) *years* (*cinnamon group*)	Duration = 16 weeks Placebo group (wheat flour, 2.5g/day) or the cinnamon intervention group (3 g/day)	Primary = FBG, PBG and HbA1c	Significantly greater decrease in FBG (P = 0.001*), and HbA1c (P = 0.011*) in the cinnamon group, but no significant effect in PBG (P = 0.055)
Curcuminoids	Na et al. [18]	China	Double-blind, placebo-controlled RCT	Demethoxy-curcumin, bisdemethoxycurcumin, sesquiterpene ketones and alcohols	Overweight/obese with T2Ds, BMI ≥ 24.0 kg/m2, FBG ≥ 7.0 mmol/L or PBG ≥ 11.1 mmol/L, current optimal therapeutic regimens lasting at least 6 months	*n 100* (*F = 50 and M = 50*) *Age = 54.72* (*SD 8.34*) *years* (*placebo*), *55.42* (*SD 6.40*) *years* (*curcuminoids*)	Duration = 3 months Placebo or a 150mg curcuminoids capsule twice daily, 30 min after breakfast and supper, respectively	Primary = FBG and HbA1c Secondary = HOMA-IR	Curcuminoids supplementation has a significantly decreased in FBG (P< 0.01*), HbA1c (P = 0.031*) and insulin resistance index (P< 0.01*) in both groups.
	Panahi et al. [41]	Iran	Double-blind, placebo-controlled RCT	Piperine, demethoxycurcumin, bisdemethoxycurcumin	Not originally receiving lipid-lowering therapy, diagnosis of MetS	*n 100* (*F = 50 and M = 50*) *Age = 43.46* (*SD 9.70*) *years* (*placebo*), *44.80* (*SD 8.67*) *years* (*complex group*)	Duration = 8 weeks Placebo or daily dose of 1g (500 mg b.i.d.) of C3 Complex (5mg piperine added to each 500mg curcumin capsule)	Primary = FBG and HbA1c	Curcuminoids supplementation caused a significant reduction of FBG (P < 0.001*) and in serum levels of HbA1c (P = 0.048*)
Ginger	Mozaffari-Khosravi et al. [19]	Iran	Double-blind, placebo-controlled RCT	N/A	T2DMs for at least 10 years, FBG < 180 mg/dL and 2 h-blood-sugar < 250 mg/dl, BMI < 40 kg/m^2^, no consumption of any supplements during 2 months	*n* 81 (F = 50 and M = 31) Age = 51.05 (_SD_ 7.70) years (placebo), 49.83 (_SD_ 7.23) years (ginger)	Duration = 8 weeks Placebo or daily 3 1-g capsules containing ginger powder	Primary = FBG and HbA1c Secondary = QUICKI index	A significant decrease of FBG in the ginger in comparison with the placebo (*P *=* 0.003*, changes = − 18.17 (_SD_ 35.82) mg/dL), in line with the variation in mean HbA1c [*P *= 0.02*, changes = − 0.4 (SD 1.2)%] and improvement of QUICKI index (*P *< 0.005*, changes = 0.02 (SD 0.01) mg/dL)
	Attari et al. [42]	Iran	Double-blind, placebo-controlled RCT	N/A	Obese women aged 18–45 years, BMI of 30–40 kg/m^2^	*n* 70 (F) Age = 34.54 (SD 7.91) years (placebo), 35.25 (_SD_ 7.30) years (ginger)	Duration = 12 weeks Placebo or 2 g ginger powder as 1 g tablets/day	Primary = FBG	Ginger supplementation significantly reduced serum glucose as compared to the baseline both in the placebo and ginger group (*P *< 0.0001*, changes = − 7.51 (SD 9.69) mg/dL)
Propolis	El-Sharkawy et al. [43]	Egypt	Parallel masked, RCT	N/A	T2DMs for at least 5 years, stable doses of oral hypoglycemic drugs and/or insulin for at least 6 months, Chronic Periodontitis	*n* 50 (F = 17 and M = 33) Age = 51.2 (_SD_ 6.5) years (placebo), 48.9 (_SD_ 8.3) years (propolis)	Duration = 6 months Placebo or Propolis 400 mg capsule daily, both groups with SRP	Primary = FBG and HbA1c	There were statistically significant changes in FBG (*P *< 0.01*) and HbA1c levels after 3 and 6 months of therapy compared with the placebo group
	Samadi et al. [44]	Iran	Double-blind, placebo-controlled RCT	N/A	5–10 years history of T2DMs, using the conventional therapy of oral medications	*n* 57 (F = 28 and M = 29) Age = 56.07 (_SD_ 9.02) years (placebo), 51.30 (_SD_ 6.57) years (propolis)	Duration = 12 weeks Placebo or propolis pills 300 mg	Primary = FBG and HbA1c Secondary = fasting insulin, insulin sensitivity, HOMA-IR, QUICKI index	Significantly decreased in the mean of FBG (*P *= 0.001*, changes = − 17.76 (SD 27.72) mg/dL), HbA1c (*P *= 0.004*, changes = − 0.77 (SD 1.34)%), fasting insulin, insulin sensitivity, HOMA-IR, QUICKI and β-cell function (all *P *< 0.05*) between the two groups
*Silybum marianum* (L.)	Gargari et al. [45]	Iran	Parallel, placebo-controlled, triple-blind RCT	N/A	Aged 25–50 years, diabetes at least 6 months, taking hypo glycaemic medications, BMI of 27–35 kg/m^2^, stable habitual diet for past 3 months	*n* 40 (F = 20 and M = 20) Age = 46.10 (_SD_ 4.30) years (placebo), 43.50 (_SD_ 5.76) years (silymarin supplement)	Duration = 45 days Placebo or 140 mg silymarin supplement three times daily with main meals	Primary = FBG	Silymarin supplement showed a significant influence in FBG (*P *< 0.003*, changes = − 17.8 (− 28.77, − 7.02) mg/dL)
	Ebrahimpour-koujan et al. [46]	Iran	Phase II-III, parallel, placebo-controlled, triple-blind RCT	N/A	Aged 25–50 years, diabetes at least 6 months, taking hypo glycaemic medications, BMI of 27–35 kg/m^2^, stable habitual diet for past 3 months	*n* 40 (F = 20 and M = 20) Age = 46.10 (_SD_ 4.30) years (placebo), 43.50 (_SD_ 5.76) years (silymarin supplement)	Duration = 45 days Placebo or 140 mg silymarin supplement three times daily with main meals	Primary = FBG Secondary = fasting insulin, HOMA-IR and QUICKI index	There was a significant reduction in the levels of fasting insulin, HOMA-IR and QUICKI index compared to the placebo group (all *P *< 0.05*)
*Aloe vera*	Zarrintan et al. [47]	Iran	Double-blind, placebo-controlled RCT	N/A	Aged 30–65 years, T2DMs for at least 6 months, taking only glucose-lowering drugs not using insulin	*n* 43 (F = 19 and M = 25)	Duration = 2 months Placebo or 1000 mg of Aloe vera supplements daily	Primary = FBG and HbA1c	No significant effect in the levels of the FBG and HbA1c
*Andrographis paniculate*	Widjajakusuma et al. [48]	Indonesia	Parallel, double-blind, placebo-controlled RCT	*Syzygium polyanthum*, maltodextrin	Aged ≥ 30 years, T2DMs, taking no other medicines, on any other hypoglycemic treatment for minimum 2 weeks before the study	*n* 54 (F = 32 and M = 22) Age = 55.25 (_SD_ 10.04) years (placebo), 53.74 (_SD_ 9.25) years (EM tablets)	Duration = 8 weeks Placebo or 450 mg EM tablets, 500 mg Met tablets (all group), twcie a day	Primary = FBG, PBG and HbA1c	There was a significant decrease in FBG (4 weeks *P *< 0.043*) and PBG (4 weeks *P *= 0.002*, 8 weeks *P *= 0.017*) in the extract supplementation group, but no significant effect in HbA1c level for 4 weeks (*P *=* 0.715*)
*Cornus mas* L.	Soltani et al. [49]	Iran	Double-blind, placebo-controlled RCT	Tribasic calcium phosphate powder	Aged 18–80 years, T2DMs for at least 2 years, HbA1C > 7% and < 10%	*n* 60 (F = 21 and M = 39) Age = 49.93 (_SD_ 6.12) years (placebo), 49.16 (_SD_ 5.62) years (Cornus mas)	Duration = 6 weeks Placebo or Cornus mas extract capsules, 2 capsules twice daily, 150 mg anthocyanins each capsule	Primary = FBG and HbA1c Secondary = fasting insulin	No statistically significant in FBG (*P *=* 0.130*, changes = − 14.63 (_SD_ 36.87) mg/dL) compared to the placebo group, but significant increase in insulin level as well as decrease in HbA1c
Daidzein	Ye et al. [50]	China	Double-blind, placebo-controlled RCT	Isoflavones	Aged 30–70 years, FBG of 5.6 − 7.0 mmol/L, a 2-h PG of 7.8 − 11.0 mmol/L, newly diagnosed diabetes	*n* 151 (F) Age = 56.3 (_SD_ 11.1) years (placebo), 56.4 (_SD_ 9.9) years (Daidzein), 57.0 (_SD_ 9.68) years (Genistein)	Duration = 24 weeks Placebo or (1) 50 mg of Daidzein, or (2) 50 mg of Genistein daily, and daily dose 10 g of soy protein isolated for all group	Primary = FBG, PBG and HbA1c Secondary = fasting insulin, postprandial insulin, HOMA-IR and QUICKI index	No significant difference in all outcomes among 3 groups at baseline, 12 weeks and 24 weeks in IGR women without any drug treatment (all *P *>* 0.05*)
Flaxseed	Javidi et al. [51]	Iran	RCT	N/A	BMI of 25–34.9 kg/m^2^, fasting serum glucose of 100–125 mg/dl, not use of insulin and other glucose lowering medications or herbal supplements for at least 3 months before the study	*n* 92 (F = 52 and M = 40) Age = 50.55 (_SD_ 11.54) years (placebo), 52.93 (_SD_ 8.9) years (20 g), 52.15 (_SD_ 9.15) years (40 g)	Duration = 12 weeks Placebo or (1) 20 g flaxseed powder daily, or (2) 40 g flaxseed powder daily	Primary = FBG Secondary = fasting insulin and HOMA-IR index	There was a significant reduction in FBG (20 g *P *= 0.002*, changes = 8.63 (13.74) mg/dL, 40 g *P *= 0.001*, changes = 10.30 (SD 16.22) mg/dL)) in all groups, HOMA-IR (*P *= 0.033*, changes = 0.27 (SD 0.65)%) in 20 g group compared to the baseline, but no significant in fasting insulin (all *P *>* 0.05*) between the 3 groups
Garlic	Atkin et al. [52]	UK	Double-blind, placebo-controlled crossover pilot RCT	N/A	T2DMs, aged 18–70 years, not treated with insulin	*n* 26 (F = 9 and M = 17) Age = 61 (_SD_ 8) years	Duration = 12 weeks Placebo or aged Garlic extract (kyolic), 4 capsules/day (1200 mg) for 4 weeks, then a 4 weeks washout period and entered the crossover arm	Secondary = HOMA-IR index	No significant effect in HOMA-IR in all groups compared to the baseline and placebo group
*Glycyrrhiza Glabra* L.	Alizadeh et al. [53]	Iran	Double-blind, placebo-controlled RCT	N/A	Aged 30–60 years, BMI > 25 kg/m^2^	*n* 64 (F = 37 and M = 27) Age = 33.6 (_SD_ 4.8) years (placebo), 36.0 (_SD_ 11.9) years (supplement)	Duration = 8 weeks Placebo or Licorice. 1.5 g daily, a low-calorie diet for both group	Primary = FBG Secondary = fasting insulin and HOMA-IR index	No changes in FBG in all groups compared to the baseline and placebo (*P *>* 0.05*), but the levels of insulin (*P *= 0.02*) and HOMA-IR (*P *< 0.01*) showed significant effect compared to the baseline
*Ginkgo biloba extract*	Aziz et al. [54]	Malaysia	Double-blind, placebo-controlled RCT	N/A	Aged 25–65 years, T2DMs for at least 1 year, with glycemic status uncontrolled by Met therapy alone	*n* 47 (F = 39 and M = 8) Age = 48.2 (_SD_ 10.3) years (placebo), 48.7 (_SD_ 9.6) years (GKB)	Duration = 90 days Placebo or GKB extract, 120 mg/capsule, in addition to usual Met dose (placebo = 1.24 (SD 0.67) g/day, GKB = 1.36 (_SD_ 0.45) g/day	Primary = FBG and HbA1c Secondary = fasting insulin and HOMA-IR index	The FBG level was significantly lower than baseline values (*P *< 0.001*), and GKB extract also significantly decreased in the fasting insulin and HOMA-IR (all *P *<* 0.05*)

Chinese medicine	First author	Country	Design	Other ingredient	Design	Relevant inclusion criteria	Subjects	Interventions	Relevant outcomes	Results
*Morus alba*	Trimarco et al. [55]	Italy	Monocentric, double-blind, cross-over, placebo-controlled RCT	Berberine, red yeast rice powder	Monocentric, double-blind, cross-over, placebo-controlled RCT	Aged 18–70 years, hypercholesterolemia not requiring statins or in statin intolerant	*n* 23 Age = 59.5 (_SD_ 6.3) years	Duration = 8 weeks Two randomized: (1) Combination A (placebo) for 4 weeks followed by 4 weeks of Combination B (Morus alba), (2) Exchange squence	Primary = FBG and HbA1c Secondary = fasting insulin and HOMA-IR index	There was a significant reduction for FBG (*P *< 0.0001*), only after treatment with the Combination B, as well as HbA1c (*P *< 0.002*), insulin (*P *< 0.006*) and HOMA-IR index (*P *< 0.006*)
*Nigella sative*	Heshmati et al. [56]	Canada	Double-blind, placebo-controlled RCT	N/A	Double-blind, placebo-controlled RCT	Aged 30–60 years, T2DMs for at least 6 months, taking anti-diabetic medications	*n* 72 Age = 47.5 (_SD_ 8.0) years (placebo), 45.3 (_SD_ 6.5) years (NS oil)	Duration = 12 weeks Placebo or NS oil capsules 3 g/day, three times a day	Primary = FBG and HbA1c Secondary = fasting insulin and HOMA-IR index	FBG changed significantly in the intervention group compared to baseline, but HbA1c, insulin and HOMA-IR changed significantly in intervention group compared to the placebo group after 12 weeks intervention
Psyllium	Abutair et al. [57]	Palestine	RCT	N/A	RCT	Aged > 35 years, newly identified T2DMs patients (maximum 1 year)	*n* 40 (F = 20 and M = 20)	Duration = 8 weeks Both groups remain regular diet, and intervention group provided with 10.5 g of psyllium soluble fiber daily	Primary = FBG and HbA1c Secondary = fasting insulin and HOMA-IR index	There was a significant effect in FBG level in the intervention group compared to the placebo group, as well as the level of HbA1c, insulin and HOMA-IR (all *P *< 0.001*)
Red ginseng	Oh et al. [58]	Korea	Double-blind, placebo-controlled RCT	N/A	Double-blind, placebo-controlled RCT	Aged 20–75 years, FBG of 5.6–7.8 mmol/l with at least two follow-up measurements	*n* 42 (F = 14 and M = 28) Age = 53.5 (_SD_ 1.9) years (placebo), 53.2 (_SD_ 1.8) years (red ginseng)	Duration = 4 weeks Placebo or three fermented red ginseng (FRG) capsules/day with 2.7 g/day	Primary = FBG, PBG and HbA1c Secondary = fasting and postprandial insulin	FBG level was reduced by FRG (*P *= 0.039*), but did not show a treatment effect when compared to the placebo. No differences in fasting insulin were found, but FRG led to a significant effect in PBG (*P *= 0.008*) and postprandial insulin (*P *= 0.040*) levels compared to the placebo.

Seventeen herbal ingredients used in Chinese herbal formulations were involved: cinnamon (n = 4), ginger (n = 2), curcuminoids (n = 2), *Silybum marianum* (n = 2), propolis (n = 2), *Andrographis paniculata* (n = 1), garlic (n = 1), ginseng (n = 1), *Ginkgo biloba* (n = 1), *Glycyrrhiza glabra* L. (n = 1), *Morus alba* (n = 1), *Nigella sative* (n = 1), flaxseed (n = 1), daidzein (n = 1), *Aloe vera* (n = 1), psyllium (n = 1), *Cornus mas* (n = 1).

The study populations included diabetes patients (n = 16), pre-diabetes (n = 3) or people with predisposing factors of diabetes (n = 5). Predisposing factors included overweight or obese populations, and those with hypercholesterolemia and metabolic syndrome. What’s more, comparisons were made against placebo (n = 22), conventional medicine (n = 1) and regular diet (n = 1).

Of the 24 studies, 23 tested the effect on FBG levels (n = 23), PBG levels (n = 4) and HbA1c levels (n = 18) as the primary outcomes. Nineteen studies reported some statistically significant reductions in the respective measures while 5 studies showed no effect on primary or secondary outcomes.

### CONSORT evaluation

Table 2 presents a summary of CONSORT quality assessment of the 24 studies included in the review. No studies met all the CONSORT criteria. Reasons for this were a lack of explanation about how the sample size was determined, how randomisation was performed, and what interventions were in place to ensure blinding; a lack of ancillary analyses to explore other influential factors; and a lack of reporting about possible harms associated with the use of the nutraceuticals.

**Table 2:**
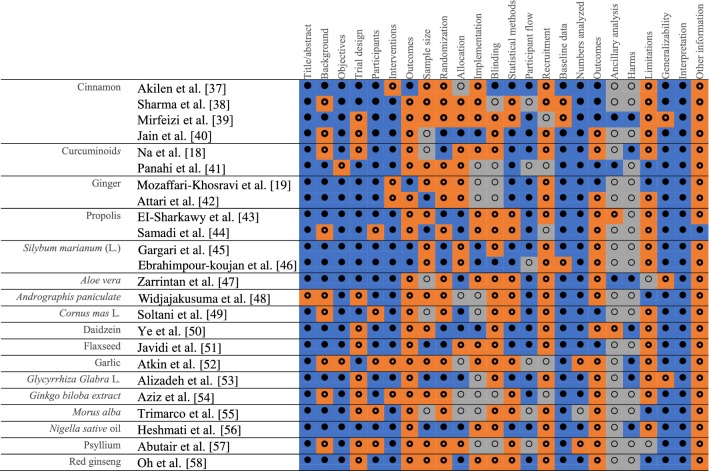
Evaluation of included treatment studies using the CONSORT checklist

full compliance;  partial compliance;  non-compliance

All 24 studies reported randomisation, but only 15 studies reported specific methods for random sequence generation, 10 studies referred to a randomisation number table with 5 of them reporting randomisation procedures; and 2 used block randomisation to determine the procedures; the remaining 3 studies used computer Random Allocation Software. Only 3 studies fully described the implementation process, including how randomisation sequences were generated, who recruited the subjects, and who intervened to implement them. Only 1 study completely reported the specific blinding procedures, and 17 referred to blinding but lacked specific procedures, such as what interventions were in place to ensure blinding. The remaining 6 studies did not mention blinding procedures. Only 7 studies reported the primary and secondary outcomes of each subgroup, including both the estimated value and precision, while the remaining 17 were incomplete statements; 2 studies presented the ancillary analyses to explore other influential factors, including subgroup analysis and calibration analysis, 3 statements were incomplete and 19 were not presented at all. Seven studies reported unintended consequences or possible harms in each subgroup, 3 of which were gastrointestinal adverse events, and the remaining 17 did not report adverse events or possible harms.

### Risk of bias

None of the 24 studies met the Cochrane criteria for low risk of bias. Six studies had an unclear risk of bias overall, and the remaining 18 had a high risk of bias. Table 3 presents a summary of the Cochrane evaluation of the 24 studies. Overall, there was a lack of detail reported to adequately assess their potential risk of bias. Only a few studies specifically reported the allocation details of how to generate and conceal the sequence during the randomization allocation.

**Table 3:** Risk of bias summary for studies included in this systematic review

First author	Sequence generation (selection bias)	Allocation concealment (selection bias)	Blinding of participants and personnel (performance bias)	Blinding of outcome assessors (detection bias)	Completeness of outcome data (attrition bias)	Selective reporting (reporting bias)	Academic bias	Sponsor bias	Overall
Akilen et al. [37]	Low	Unclear	Low	Low	Low	Unclear	High	High	High
Sharma et al. [38]	Unclear	Low	Low	Low	High	High	Low	Unclear	High
Mirfeizi et al. [39]	Unclear	Unclear	High	High	Low	Unclear	Low	High	High
Jain et al. [40]	Low	Low	Low	Low	Low	Unclear	Low	Low	Unclear
Na et al. [18]	High	Low	Low	Low	Low	Unclear	Low	Low	High
Panahi et al. [41]	Unclear	Unclear	Unclear	Unclear	Unclear	Unclear	High	High	High
Mozaffari-Khosravi et al. [19]	Low	Unclear	Low	Unclear	Low	Low	Low	High	High
Attari et al. [42]	Low	Low	Unclear	Unclear	Low	Unclear	High	High	High
El-Sharkawy et al. [43]	Low	Low	Low	Unclear	Low	Unclear	Low	High	High
Samadi et al. [44]	Low	Unclear	Unclear	Unclear	Low	Unclear	Low	High	High
Gargari et al. [45]	Low	High	Low	Low	Low	Low	High	High	High
Ebrahimpour-koujan et al. [46]	Low	High	Low	Low	Low	Low	High	High	High
Zarrintan et al. [47]	Low	Unclear	Low	Low	Low	Unclear	High	High	High
Widjajakusuma et al. [48]	Unclear	Unclear	Unclear	Unclear	Unclear	Unclear	Low	Low	Unclear
Soltani et al. [49]	Unclear	Unclear	Low	Low	Low	Unclear	High	High	High
Ye et al. [50]	Low	Low	Low	Low	Unclear	Low	Low	Low	Unclear
Javidi et al. [51]	Unclear	Unclear	Unclear	Unclear	Low	Low	Low	Low	Unclear
Atkin et al. [52]	Unclear	Unclear	High	High	Low	Unclear	Low	Low	High
Alizadeh et al. [53]	Low	Low	Low	High	Low	Unclear	Low	High	High
Aziz et al. [54]	Unclear	Unclear	Unclear	Unclear	Unclear	Low	High	High	High
Trimarco et al. [55]	Unclear	Unclear	Unclear	Unclear	Unclear	Unclear	Low	Unclear	Unclear
Heshmati et al. [56]	Low	Low	Low	Low	Low	low	Low	High	High
Abutair et al. [57]	Low	Unclear	Unclear	Unclear	Low	Unclear	Low	Low	Unclear
Oh et al. [58]	Low	Unclear	Unclear	Unclear	Low	Low	High	High	High

Low—low risk of bias; Unclear—unclear risk of bias; High—high risk of bias

Meanwhile, many studies lack a report on how blinding of participants, personnel, and outcome assessors was performed. Because of this, eight studies have unclear performance bias and two have high performance bias. Similarly, ten studies have unclear detection bias and another three have high detection bias after evaluating the blinding of outcome assessors. To ensure the data integrity, five studies showed the unclear attrition bias, and one showed high bias, the most important of which was the lack of P-values compared to baseline data and lack of the difference between pre- and post-intervention changes. The bias of the selective reporting in 12 studies were unknown, and another study had a high reporting bias. Seven studies have academic high risk of bias. Only six studies had low sponsor bias and the rest had high and unclear risk of bias.

## Discussion

This systematic evaluation of the literature identified evidence to support the use of nutraceuticals containing TCM to improve FBG, PBG, and HbA1c levels. However, shortcomings in the quality of the study design and reporting and potential bias were identified. While this may partly be due to the current methodological shortcomings of RCT study designs and the complex characteristics of TCM, the results prompt further exploration about how to improve the methodology to build a stronger evidence-base that supports the use of TCM as nutraceuticals in diabetes management. There is also a clear need for training researchers in appropriate trial reporting as many of the gaps identified may have been closed if a more comprehensive report of their study was provided.

### Nutraceuticals as an adjunct therapy

Integrating conventional and complementary medicines with integrated healthcare has been a global phenomenon in the past 2–3 decades [59, 60]. This trend is reflected by the constant demands for complementary medicines from patients. In 1998, a cancer centre in the United States established a multidisciplinary team to meet patients’ needs for integrating conventional medicines and complementary medicines [61]. As of 2011, there was also a survey in Japan showed that almost 90% of doctors had forms of complementary medicines in their prescriptions [62]. A number of examples of using TCM as a complementary therapy to promote the self-management of diabetic patients are identified in the literature. In vitro studies suggested that cinnamon decreased the activity of intestinal maltase, sucrase, and pancreatic alpha-amylase and that this effect was additive with acarbose [63]. Some clinical research evaluated the effects of garlic when used in combination with antidiabetic drugs and showed that taking a specific garlic powder product 300 mg three times daily in combination with metformin for 24 weeks could reduce fasting blood glucose approximately 70% more than with metformin alone in patients with type 2 diabetes [64]. Some TCM ingredients in this review have been shown to reduce levels of glucose and HbA1c or elevate plasma insulin levels through evidence from human or animal models, including curcuminoids, ginger, *Aloe vera* [65–67]. Other ingredients were used traditionally as antidiabetic agents, such as *Cornus mas* L., daidzein genistein, *Nigella sative* oil [68–71]. These provide evidence that some diabetic patients have taken nutraceuticals containing TCM as a complementary treatment programme for a long time, however, there is still not enough evidence to put forward practical clinical recommendations. Combined with this assessment of the quality of RCT reports, the overall quality of current clinical trials does not sufficiently ensure the long-term safety and effectiveness of nutraceuticals containing TCM as complementary therapies.

### Major areas of improvement in the RCT design

Taking the evaluation results into account, there are some limitations in the studies included in this review. Firstly, the overall quality of the studies was poor. Whether through the CONSORT checklist statement or the Cochrane risk-of-bias tool, explanations for generating randomisation and blinding are necessary to avoid RCTs bias. However, only half of the studies reported randomisation methods generated via random sequences and only six studies (25%) reported the detailed blinding procedures, which may lead to perceptions of performance bias and detection bias [72]. Therefore, it is necessary to fully report the specific details of randomization and blinding measures in accordance with the CONSORT checklist in the RCTs. Meanwhile, the Chinese Concort Group for TCM has submitted precise reports of TCM interventions and outcome measures in order to improve the quality of TCM research [73]. An RCT guideline that is specific for nutraceuticals combined the CONSORT 2010 checklist with the reports of TCM may be a better choice for the future research.

Secondly, most sample sizes in the included studies were small. Seven studies (29.2%) had a small sample size of less than 50, 11 studies (45.8%) had a sample size between 50 and 100, and the others (25%) ranged in sample size between 100 and 200. This situation often leads to a lack of statistical ability to properly estimate the effect of treatment, while also overestimating the risk of intervention benefit. Most of the subjects in this review required patients with diabetes when recruiting in the hospital, while diabetic patients needed long-term medication or insulin treatment. It was difficult to determine whether or not patients maintained the original drug regimen, or added the nutraceutical supplement, or executed intervention after entering the washout period.

Thirdly, all periods of the reported studies ranged from 4 to 24 weeks, and the periods of 10 studies were less than or equal to 8 weeks. While the short-term design was sufficient to demonstrate some immediate effects of the nutraceuticals on reducing blood glucose level, the long-term effects on the overall management of diabetes were not fully investigated. For example, the duration of the RCTs is important to detect reliable HbA1c changes. HbA1c can reflect a long-term average blood glucose level, and the change mostly depends on red blood cells’ life span, which varies from person to person. In addition, it has been proven that it usually takes 1 month for HbA1c changes to reach its 50% maximum capacity, and 2 months for 80% changes [74]. Therefore, performing a trial of at least 2 months would cover the subject’s difference in red blood cell life span. In addition, the sustainable efficacy issues of long-term use of nutraceuticals containing TCM are also worthy of further consideration.

Although many of the issues surrounding the safety and effectiveness of nutraceuticals and TCM used in modern society have not been effectively addressed, improving the quality of RCT reports can provide a larger evidence base. Therefore, the design of future RCTs should pay more attention to randomisation and blinding measures, increase sample size and duration of trials, completed reporting and analysis of adverse reactions.

### Other safety concerns regarding the use of nutraceuticals in managing diabetes

Many users believe that it is not necessary to consult their healthcare provider before taking nutraceuticals especially when these products were not sold as drug in the market or defined as medicines by the authority such as the US Food and Drug Administration [75]. However, with at least 23,000 emergency medical visits per year being related to nutraceuticals or dietary supplements in the US between 2004 and 2013, nutraceuticals are certainly not as safe as many assume [76]. At present, the most common safety issues for nutraceuticals containing TCM are the interactions of the bioactive components themselves in TCM (TCM–TCM), and the interactions between nutraceuticals containing TCM and conventional medicines.

#### TCM–TCM interactions

Traditional medicine practitioners and medical professionals generally believe that ingredient herbal formulations are usually effective because of their long history of successful use [77]. However, the interaction of the TCM ingredients may result in a change in the efficacy of the formulations, and may even enhance or ameliorate adverse effects [77]. Many of the latest publications are now studying the interaction of TCM ingredients. On the one side, Shoba et al. [78] has long shown in theirs’ research that piperine can enhance the serum concentration, absorption and bioavailability of curcumin both in rats and humans without side effects. Studies by Hassan et al. [79] showed that cinnamon-ginger mixture extract improved the glucose levels, serum triglycerides and other biochemical indicators in gestational diabetic rats. On the other side, the methanolic extract of *Panax ginseng* added to aristolochic acid-treated proximal tubule epithelial cells (HK-2 cells) was found to induce renal epithelial cells to accelerate apoptosis [80]. Considering the possibility of interactions, positive or negative, among the TCM ingredients, the overall safety deserve more attention even if each individual nutraceutical has been shown to be effective.

#### TCM-conventional medicine interactions

The increased global use of nutraceutical containing TCM, and a lack of accurate information about nutraceutical containing TCM being available to healthcare providers and patients, have increased concerns about the interactions of TCM and conventional medicines [81]. A recent review of studies on herb-drug interaction published between 2000 and 2014 showed that the number of such reports continued to increase and most studies examined the pharmacokinetic interactions through in vivo studies, in vitro studies, and review studies [82]. More studies, especially clinical studies focusing on pharmacodynamics, are warranted to fully investigate the safety profile of nutraceuticals containing TCM. Only 7.4% of the publications analysed the interaction of TCM with the treatment of diabetes drugs, and most current research focuses on tumour and circulatory diseases [82]. Studies have shown that there is a positive or negative interaction between TCM and conventional medicines to increase or decrease the efficacy of traditional drugs [83]. Ashraf et al. [84] have confirmed that the combination of appropriate garlic extract (*Allium sativum* L.) and metformin showed a significant reduction in fasting blood glucose compared to using alone metformin (*P *<* 0.05*), and their study also reported that the interaction caused a significant decrease in the mean total cholesterol level (*P *<* 0.05*). In contrast, a case study reported in 2015 showed that the combination of cinnamon and statins has the potential for significant liver damage and it should be discouraged [85], what’s more, in the theoretical point of view, concurrent use of cinnamon with blood sugar-lowering agents may have additive effects and increase the risk of hypoglycemia [86].

#### Potential adverse reactions

Studies have shown that in RCTs, adverse reactions of TCM for treatment are rarely observed compared with placebo groups [87]. However, RCTs cannot reliably detect rare adverse events or have a significant incidence of latency time [88]. Di Lorenzo et al. [89] pointed out in a systematic review that 32% of the adverse reactions of plant-based nutraceuticals and preparations were caused by soybean (*Glycine max*) and licorice (*Glycyrrhiza glabra*), and the two are included in this evaluation. Seven of twenty-four studies reported adverse reactions, and three were gastrointestinal reactions in this evaluation, but none of them reported the cause of the adverse reactions. Especially for chronic diseases, adverse reactions are caused not only by conventional causes such as pharmacokinetics, but also by exposure to other compounds (alcohol, nicotine) and heterogeneity factors in nutraceuticals that confuse them with mechanism of clinical adverse reactions during the long-term administration. Therefore, future RCT models for nutraceuticals should take into account the need to assess the risks of drug-herb interactions and the adverse reactions associated with long-term use.

Overall, paying attention to the quality of RCT reports is critical to improving the evidence-base for nutraceuticals containing TCM for diabetes management. In addition, clinical data about the long-term safety (including case reports, post-marketing surveillance studies, etc.) is also important for developing a more complete safety profile as nutraceuticals compsing of TCM may be used continuously in diabetes management.

## Limitation

This systematic review based on the existing standards for literature retrieval, evaluation and data synthesis, presents a complete view of the nutraceuticals containing TCM for diabetes management and the quality of its RCT reports. Nevertheless, our study has some limitations. Firstly, 16 of the 24 studies were conducted strictly for diabetic patients, with each of them employing different study design. To further develop the evidence base about nutraceuticals containing TCM in diabetic management, more rigorously designed clinical trials are needed. Secondly, the publication bias cannot be completely ruled out, that is, regardless of the methodological quality, the data showed according to the results are unlikely to fully present the negative findings. What’s more, we only reviewed the TCM herbs listed in the Chinese Pharmacopoeia, but the TCM mentioned in this study is also included in the pharmacopoeia of other countries, and can expand the scope of TCM in future research to analyze more RCT reports. Another limitation in this study was the exclusion of Chinese publications. In the preliminary literature search, a literature search at the National Knowledge Infrastructure CNKI using the same search strategy was conducted but we were not able to identify any Chinese publications which investigated the evidence base for the efficacy about TCM used as nutraceuticals for diabetic management. To further illustrate the evidence base about the efficacy of TCM which can potentially be used as a nutraceutical, another larger-scale systematic literature review is underway which aims to identify TCM shown to be effective in diabetic management and can be defined as nutraceuticals according to the regulations in different countries.

## Conclusion

There is some evidence obtained from clinical trials to support a role for nutraceuticals containing TCM in the management of diabetes. However these findings are tempered by the overall poor quality of trial reporting and therefore caution is required in translating these findings to clinical practice and standard care. More rigorous long-term clinical trials to investigate both safety and efficacy of TCM nutraceuticals are warranted and authors are encouraged to follow detailed guidelines for reporting RCTs.

## Data Availability

All data are fully available without restriction.

## Abbreviations

TCM: traditional Chinese medicine
RCTs: randomized controlled trials
FBG: fasting blood glucose
PBG: postprandial 2-h blood glucose
HbA1c: glycated hemoglobin
T1DM: type 1 diabetes mellitus
T2DM: type 2 diabetes mellitus
ADA: American Diabetes Association
IGT: impaired glucose tolerance
IFG: impaired fasting glucose
MetS: metabolic syndrome (MetS)
